# The Shape of the Foveal Avascular Zone: When a Circle Isn't Round

**DOI:** 10.1167/tvst.12.6.26

**Published:** 2023-06-28

**Authors:** Jenna Grieshop, Mina Gaffney, Rachel E. Linderman, Robert F. Cooper, Joseph Carroll

**Affiliations:** 1 Joint Department of Biomedical Engineering, Marquette University and Medical College of Wisconsin, Milwaukee, WI, USA; 2 Ophthalmology & Visual Sciences, Medical College of Wisconsin, Milwaukee, WI, USA; 3 Wisconsin Reading Center, Madison, WI, USA; 4 Cell Biology, Neurobiology & Anatomy, Medical College of Wisconsin, Milwaukee, WI, USA

**Keywords:** foveal avascular zone, biomarker, OCTA

## Abstract

Numerous metrics are used to analyze the morphometry of the foveal avascular zone (FAZ). Two such metrics, roundness and circularity, have recently shown a marked growth in their use. However, there have been inconsistencies across studies with respect to how these metrics are defined, as well as the algorithms used to calculate them. In some cases, the exact definition or algorithm is not disclosed. These issues significantly limit the translational utility of these biomarkers. We simulated FAZ shapes as circles or ellipses of differing aspect ratios and evaluated roundness and circularity with two commonly used approaches for FAZ analyses. Differing shape analysis algorithms produced conflicting results even with identical equations, and differing metric definitions produced incongruent results. Therefore caution should be used when comparing FAZ circularity and roundness metrics across studies, especially in the absence of detailed information about the algorithms used.

**Translational Relevance:**

Quantitative assessment of OCT-A images includes evaluating circularity and roundness of the FAZ. Inconsistent or inaccurate mathematical definitions of these metrics impacts their utility as biomarkers and impairs the ability to combine and compare results across studies.

## Introduction

Optical coherence tomography angiography (OCTA) is a noninvasive imaging modality that enables direct visualization of the retinal vasculature, including the foveal avascular zone (FAZ).^1^^,^^2^ Multiple studies have characterized the size and shape of the FAZ in individuals with normal vision,^1^^,^^3^^–^^9^ and others have shown that the FAZ is altered in retinal pathologies such as diabetic retinopathy,^3^^–^^5^ sickle cell retinopathy^10^^,^^11^, and retinopathy of prematurity.^12^^,^^13^ Quantitative metrics that have been examined as potential FAZ biomarkers for these and other diseases include area, perimeter, circularity, and roundness.^1^^,^^3^^–^^5^^,^^8^^,^^9^^,^^11^^,^^14^ Although area is perhaps the most common metric used to describe the FAZ, the enormous normal variation in the size of the FAZ^1^^,^^9^ may limit its ability to signify pathology in cross-sectional screening applications.^3^ The regularity of the overall shape of the FAZ (measured as roundness or circularity) may enable more sensitive indication of disease because of less variation in the healthy population.^1^ Central to the use of these metrics as FAZ biomarkers is an understanding of their mathematical definitions, as this not only impacts the sensitivity of the metric but also affects the ability to combine or compare data across studies.

With respect to roundness and circularity, there are inconsistencies in methodology across studies. Roundness and circularity definitions, as well as the algorithms and methods used to calculate these metrics, have been contradictory and frequently undisclosed throughout literature.^1^^,^^3^^–^^5^^,^^9^^,^^15^ Even when included, inaccurate formulas have been reported.^1^^,^^4^^,^^16^ Roundness and circularity are often interpreted ambiguously and, without precise definitions, can be mistakenly used interchangeably or inconsistently between studies.^1^^,^^3^^–^^5^^,^^9^^,^^14^^,^^15^ Similarly, the use of different algorithms to calculate these metrics can introduce conflicting results based on the techniques or software being used.

**Figure 1.**
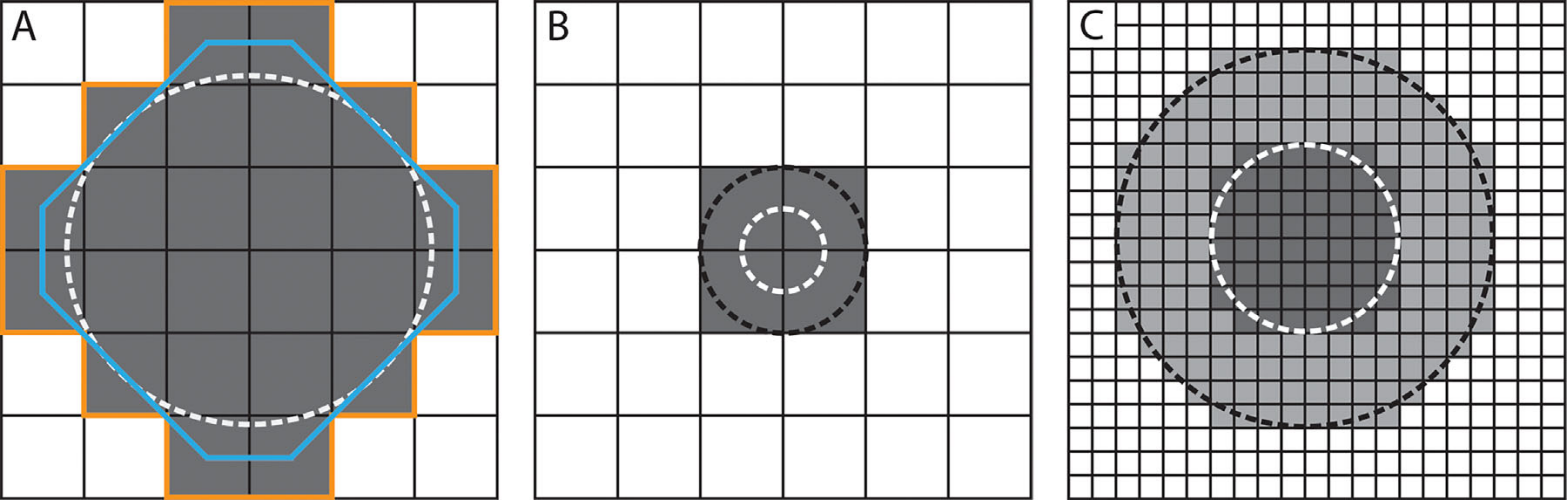
Effect of pixelation on defining shape perimeter. (**A**) An example of two algorithms measuring conflicting perimeter values for the same shape. The *white dashed circle* represents true shape perimeter, the *gray squares* are the pixelized representation of the shape, and the *orange* and *blue lines* are two theoretical algorithmic methods of measuring the shape's perimeter. (**B**) An example of low image resolution. Both the *dashed black* and *white shapes* are represented by the same four pixels, although the *black shape* is double the size of the *white shape*. (**C**) The same shapes as Panel B, although upsampled by a factor of 8. With higher resolution the difference in size between the shapes becomes detectable.

Several distinct methods are used in shape tracing and measuring perimeter—a value weighted heavily in circularity calculations. The precision of this measurement varies because of the method of rounding and pixel handling used by the algorithm; thus incongruent results may be observed for identical shapes, depending on the algorithm used, as well as the image scale in pixels, as illustrated in Figure 1A. Therefore it is crucial to either use the same algorithms and methods across studies or to fully disclose this information so comparisons can be made across studies. This includes specifying whether the OCTA image was resampled in any way.

Although low-image resolution can contribute to pixel-handling inconsistencies, it can also cause true border loss independently from the algorithm used, as seen in Figure 1B. Sub-pixel measurements are needed to avoid both pixel handling and low image resolution issues and achieve the most accurate results as they limit erroneous pixel rounding by using exact coordinates of the shape border. A well-known instance of a similar nature is the coastline paradox, in which the length of a coastline depends on the size of the smallest unit of measurement considered. The length of the coastline increases as the unit of measurement becomes smaller, as it can account for smaller features of the contour.

To promote cognizance of the limitations of unclear definitions, inconsistent algorithm modalities, and the lack of disclosure present in current literature, we present data simulations to compare computations of shape metrics methods with sub-pixel accuracy, as well as two commonly used methods for FAZ analysis, MATLAB *regionprops* (Mathworks, Natick, MA) and ImageJ *Analyze Particles.*^17^

## Methods & Results

### Definitions of Roundness and Circularity

**Figure 2.**
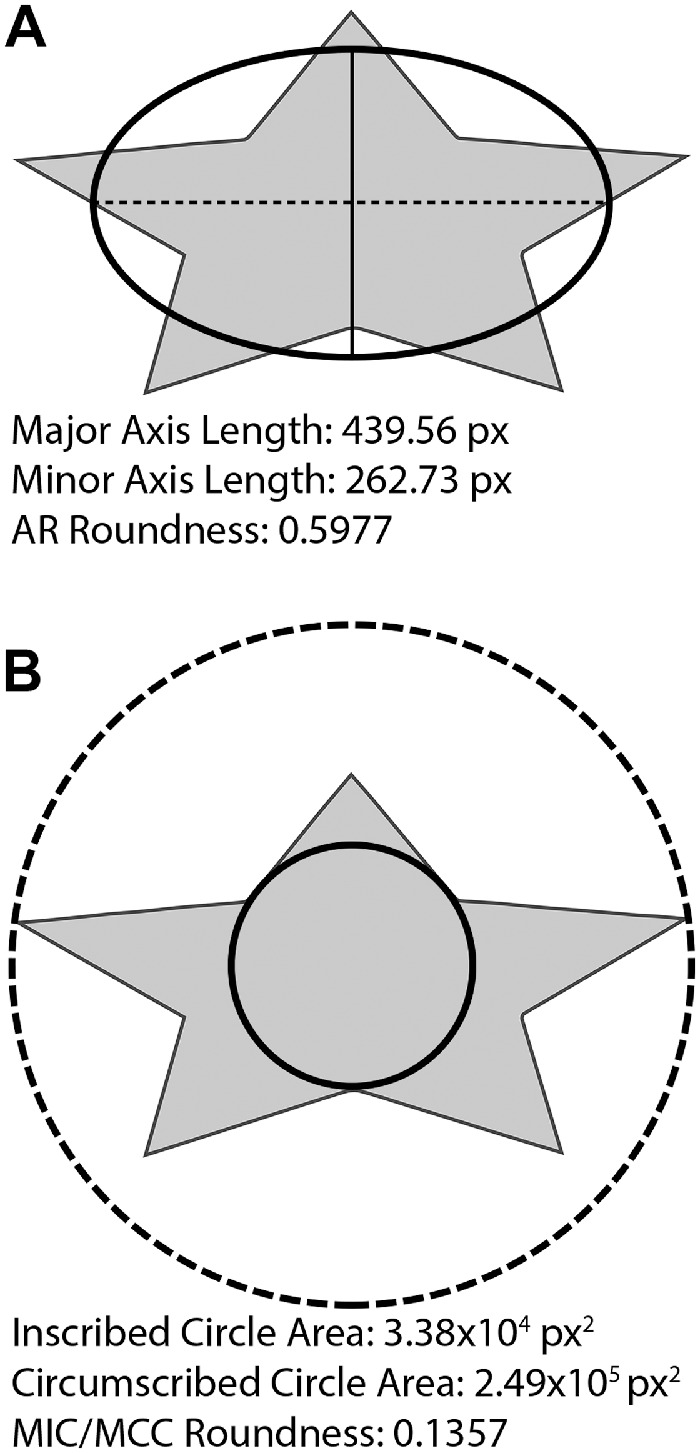
Visualization of AR and MIC/MCC roundness metrics for the same shape. AR roundness (**A**) requires major (*dashed*) and minor (*solid*) axes derived from the best-fit ellipse. The axis ratio is used as a measure of roundness. MIC/MCC roundness (**B**) requires a minimum circumscribed *circle* (*dashed*) and a maximum inscribed *circle* (*solid*). The ratio of the area of the *circles* is used as a separate measure of roundness.

It is essential to first define what is meant by circularity and roundness. Roundness has at least two definitions; here we examined aspect ratio (AR) roundness and maximum inscribed circle to minimum circumscribed circle ratio (MIC/MCC) roundness. AR roundness, defined as:

$$\begin{array}{c} AR\;Roundness=\frac{4*\;Area}{\pi *Major\;Axis^{2}}=\;\frac{Minor\;Axis}{Major\;Axis} \end{array}$$

measures how close the proportions of the shape are to a circle, and the major and minor axis are derived from the best-fit ellipse. As the shape becomes more circular, AR roundness will approach a value of 1. MIC/MCC roundness, defined as

$$\begin{array}{ccc} & & \;MIC/MCC\;Roundess\\ & & \;=\frac{Maximum\;Inscribed\;Circle\;Area}{Minimum\;Circumscribed\;Circle\;Area} \end{array}$$

describes a ratio of the area of two circles, the largest inscribed circle that fits within the object being measured and the smallest circumscribed circle that fully contains the object being measured. As the shape becomes more circular, MIC/MCC roundness will also approach a value of 1. A visual representation of both roundness metrics is shown in Figure 2.

Circularity (sometimes casually interchanged with roundness) quantifies irregularities in shape border, because it relies heavily on the perimeter of the shape. Here, we and others define circularity as

$$\begin{array}{c} Circularity=4\;*\;\pi \frac{Area}{Perimeter^{2}} \end{array}$$

It is worth noting that the International Standard of Organization ISO 9276-6:2008 document defines circularity as

$$\begin{array}{c} Circularity=\;\sqrt{4\;*\;\pi \frac{Area}{Perimeter^{2}}} \end{array}$$

however, the majority of OCTA literature uses the first equation, so we have used that in our subsequent analysis.^1^^,^^14^ Additionally, the mathematical relationship between AR roundness and circularity can be found by equation manipulation of the two definitions, and is defined as

$$\begin{array}{c} Circularity=\;\frac{AR\;Roundness*\;\pi^{2}*\;Major\;Axis^{2}}{Perimeter^{2}} \end{array}$$

### Shape Simulations

To examine the effects of contrasting definitions and algorithms used, various shapes were rendered in MATLAB to simulate FAZs of differing sizes and morphologies. Thirty-seven circles with radii ranging from two to 1000 pixels were created. For each circle in the set, three ellipses of the same area were created, with aspect ratios of 10:4, 10:6, and 10:8. All circle and ellipse sets were generated using trigonometry, with a 300-point sampling rate, *t*, to generate the *x* and *y* coordinates of the points along the perimeter as defined below.

$$\begin{array}{c} x=radius*\cos\left(t\right)\;\text{and}\;y=radius*\sin\left(t\right) \end{array}$$

All data and calculations were collected through a single automated MATLAB script capable of connecting to ImageJ (https://github.com/AOIPLab/Circularity_vs_Roundness_FAZ). Measurement data were obtained for each shape using the built-in *regionprops* MATLAB function and ImageJ *Analyze Particles* (ImageJ>Analyze>Analyze Particles). MATLAB *regionprops* was called to measure area, two perimeter values (to account for the new and old algorithms used to calculate perimeter), circularity, major axis length, and minor axis length for each shape. These values were saved, and AR roundness was calculated by setting a ratio between the minor and major axis length. ImageJ *Analyze Particles* was called to measure area, perimeter, circularity, roundness, major axis length, and minor axis length. *Analyze Particles*’ roundness definition is equivalent to the AR roundness definition here.

MIJ^18^, a Java package to connect MATLAB and ImageJ, was used in combination with Fiji^19^ to access and run *Analyze Particles* from the MATLAB script. To do this, an instance of ImageJ was created, navigated through, and terminated using MIJ methods and macro commands in the MATLAB script. To run *Analyze Particles*, a mask of the shape was opened in the ImageJ instance, and the image was thresholded using default parameters (Image>Adjust>Threshold). Area, perimeter, shape, and fit were selected as measurements for *Analyze Particles* (Analyze>Set Measurements). The results table generated by *Analyze Particles* in ImageJ was saved into MATLAB.

In addition to *Analyze Particles* and *regionprops*, mathematical computations performed in MATLAB included calculating theoretical values, calculating the sub-pixel perimeter using the distance formula and shape coordinates, and using MATLAB *polyarea* to measure the sub-pixel shape area. The MATLAB functions *ExactMinBoundCircle*^20^ and *max_inscribed_circle*^21^ were used to determine the diameters of the best-fit circles to calculate MIC/MCC roundness.

While *regionprops* and *Analyze Particles* resulted in identical shape area values, the *polyarea* function resulted in better agreement with theoretical values (Mann-Whitney U = 2672, *P* < 0.0001). Similar to *polyarea*, the sub-pixel perimeter was most accurate to theoretical values (Kruskal-Wallis H(4) = 371.0, *P* < 0.0001). The Table describes the percent difference between theoretical calculations and area and perimeter measurement methods along with their mathematical relationships in which theoretical value is represented as *y* and measurement value is represented as *x*.

**Table.** Percent Difference and Mathematical Relationships Between Theoretical and Measured Values for Area and Perimeter Methods

	*polyarea* Area	*regionprops/Analyze Particles* Area	Coordinate Perimeter	*regionprops* Old Perimeter	*regionprops* New Perimeter	*Analyze Particles* Perimeter
Min % Difference	0.0071	2.0792 × 10^−5^	0.0015	0.0501	0.0033	0.0058
Mean % Difference	0.0074	0.9472	0.0018	3.3208	4.6467	4.2218
Max % Difference	0.008	22.7453	0.0024	40.5697	34.9431	18.0559
Equation	y = 0.9999x −3 × 10^−6^	y = 0.9999x −2.236	y = 1x −3 × 10^−8^	y = 0.9489x +2.7597	y = 1.0002x +2.7597	y = 0.9489x +1.6507

Although *regionprops* and *Analyze Particles* selected best-fit ellipses for the shapes with differing methods to find AR roundness, both ultimately calculated identical values of AR roundness. The relationship between AR roundness and MIC/MCC roundness for circles and ellipses resulted as

$$\begin{array}{ccc} & & \;MIC/MCC\;\;Roundness\\ =1.0144*AR\;Roundness^{1.9346}\;\left(R^{2}\;=\;0.9884\right). \end{array}$$

It must be noted that the smallest shape diameter in which this relationship holds is six pixels. The relationship between *regionprops* and *Analyze Particles’* calculations of circularity for circles and ellipses resulted as

$$\begin{array}{ccc} & & \;Analyze\;Particles\;circularity=\\ 0.8889*regionprops\;circularity^{0.965}\;\left(R^{2}\;=\;0.9881\right). \end{array}$$

This relationship only holds for shape with diameters of at least 16 pixels.

It is necessary to note that although the pixel resolution of FAZ images can vary, it can be increased by upsampling the image before segmentation and analysis. This is important to consider because pixel error can substantially affect accuracy of results for small shapes,^15^ and the FAZ pixel diameter can be increased to fit into the relationships mentioned above. Figure 1C depicts an example of upsampling.

### Impact on Real FAZ Measures

In addition to simulated shapes, OCTA images from a previous study were used to further investigate the impact of differing algorithms and definitions.^9^ The OCT images were acquired with the AngioVue OCTA system (Optovue Inc., Fremont, CA) and the angiogram images were extracted using a slab of the superficial plexus layer (between 3 µm below the internal limiting membrane to 16 µm above the inner plexiform layer) 304 × 304 pixels in size. The images were of the right eye from 175 participants with no known ocular or systemic pathologies and were measured through a modified section of the MATLAB script described earlier. The area of the FAZs, measured with *polyarea,* ranged from 0.07 mm^2^ to 0.66 mm^2^ with an average of 0.28 mm^2^ and standard deviation of 0.1 mm^2^. Perimeter measured via the distance formula ranged from 1.14 mm to 3.69 mm with an average of 2.24 mm and standard deviation of 0.43 mm. As in the simulation, area, perimeter, and circularity results varied across the algorithms. Likewise, AR roundness values calculated using *regionprops* and *Analyze Particles* were the same despite having different major and minor axis lengths. Differences between the measurements were calculated by

$$\begin{array}{c} \%\;\mathrm{Difference}=\frac{\left|a-b\right|}{\left(a+b\right)÷2}\times 100. \end{array}$$

**Figure 3.**
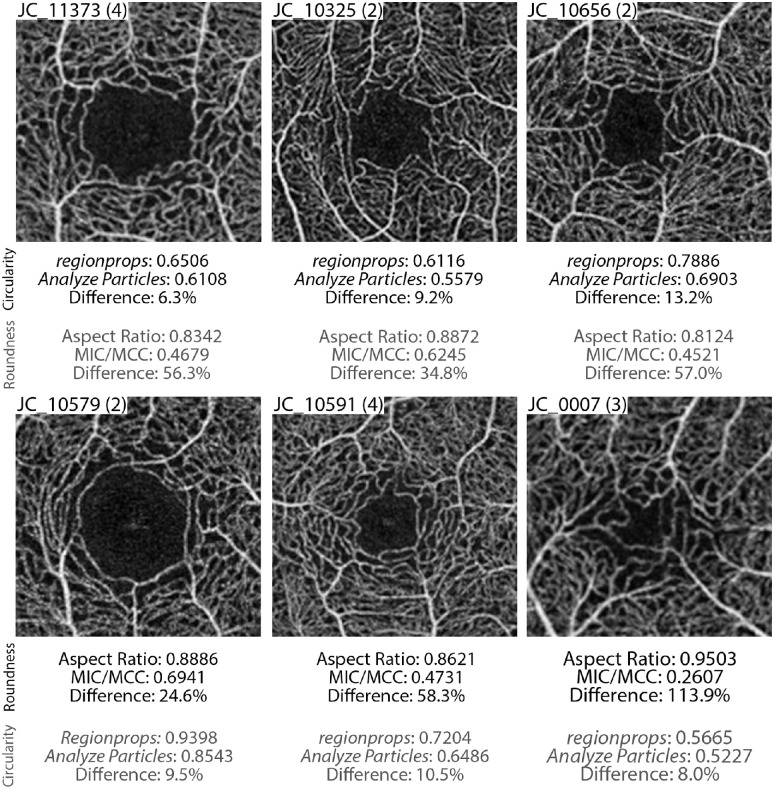
Example OCTA images with FAZs having the lowest, average, and highest measured differences in circularity (*top section*) and roundness (*bottom section*). Images shown were generated by averaging individual OCTA images to increase signal to noise ratio, with the number of frames averaged shown in parentheses. Circularity differences were computed using MATLAB *regionprops* and ImageJ *Analyze Particles,* whereas roundness was computed using AR and MIC/MCC as described in the text. For completeness, circularity and roundness values are provided for all six images.

**Figure 4.**
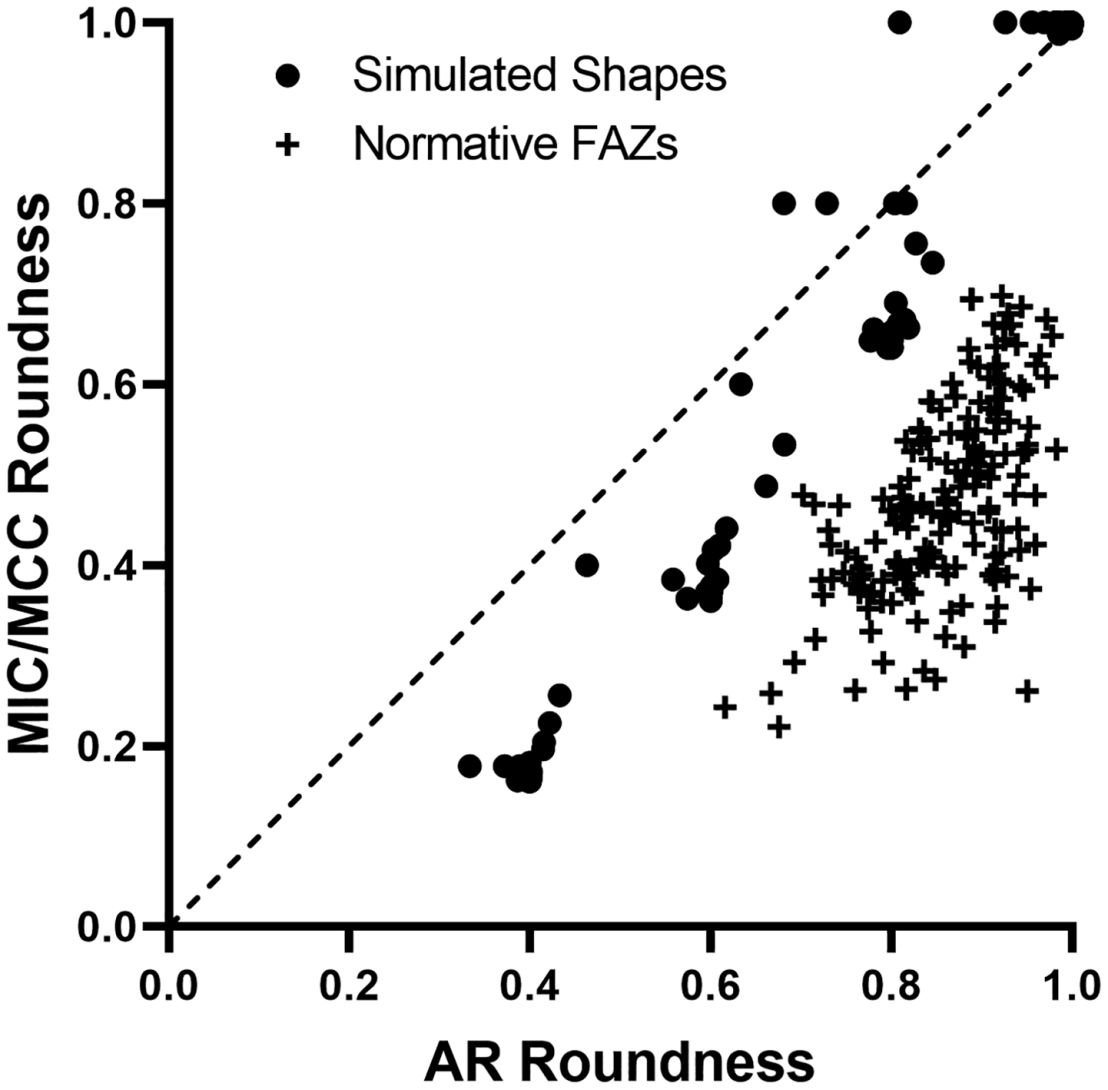
Relationship between AR and MIC/MCC roundness for simulated shapes (*filled circles*) and real OCTA images of the FAZ (*crosses*). A 1:1 relationship is represented by a *dashed line*.

Figure 3 highlights FAZs that resulted in lowest, average, and highest disagreement between circularity (top row) and roundness (bottom row) measurements. Unlike AR roundness values, *regionprops* and *Analyze Particles* generated discordant circularity values, with differences ranging 6.3% to 13.2% with an average of 9.2% and 1.3% standard deviation. Between AR roundness and MIC/MCC roundness, the percent difference, independent of algorithm used, was 24.6% to 113.9% with an average of 59.4% and 17.6% standard deviation. For all FAZs, AR roundness was greater than MIC/MCC roundness (Fig. 4). Similarly, 131/148 of the simulated shapes had AR roundness values greater than the MIC/MCC roundness value (Fig. 4). This is due to AR being derived from the best-fit ellipse, so minor irregularities in the FAZ border have less impact, whereas the MIC/MCC roundness value is more significantly impacted by small irregularities in the FAZ border (as explained in Fig. 2). When assessing trends of the differences versus the FAZ shape, we found that as the average roundness value increased, the difference between AR roundness and MIC/MCC roundness decreased (*r* = −0.7463, 95% confidence interval = −0.8055 to −0.6725, *P* < 0.0001). In contrast, as the average circularity value increased, the difference between circularity values derived from *regionprops* and *Analyze Particles* increased (*r* = 0.3966, 95% confidence interval = 0.2637 to 0.5146, *P* < 0.0001), although the effect was smaller. These relationships demonstrate a non-uniform offset between methods, further illustrating the challenge of comparing results derived from different methods.

## Discussion & Conclusion

Here we examined the challenges of quantifying and defining roundness and circularity metrics as potential FAZ biomarkers. It is important to note that there are other sources of error beyond those examined here including intraobserver and inter-observer errors in segmentation, as well as the use of different imaging devices and scan sizes.^22^ Although the change induced by different calculation methods may be small relative to some of these other sources of error, they do limit the ability to combine data across studies.

Upcoming artificial intelligence– and machine learning–based approaches of analysis or disease classification may eventually render metrics like these less important. However until then, they have an important role in characterizing retinal and systemic diseases, as well as monitoring disease progression and therapeutic response. Circularity and roundness will need to be further analyzed, especially because they describe irregular shapes, to assess sensitivity and the viability of these as biomarkers.

When choosing between MATLAB *regionprops* or ImageJ *Analyze Particles* to analyze shapes, our analysis does not suggest that one is necessarily more accurate than another, just that there are differences. Choosing which approach to use will factor into many considerations. For example, MATLAB has extensive documentation, and changes to algorithms or formulas are generally well tracked; however it is a paid subscription application. Although ImageJ is a free open-source software, it does not have the same level of documentation and it may be difficult to find information about algorithm changes. As for area and perimeter calculations, we recommend using MATLAB *polyarea* and the distance formula using the shape coordinates.

Many considerations must be realized when comparing data between studies. Terms may not be defined the same way, and if they are, the methods used to derive them may not produce comparable results. Therefore future studies should disclose in full the metric definitions, as well as the algorithms used. It is also important to take device software into consideration. As these analyses become integrated with software packages, it is important that the underlying formulas used are made transparent to clinicians and researchers. Without this information, it may be difficult for data to be accurately or reliably compared across studies. These shortcomings present in current literature may delay progress in advancing reliable biomarkers for the FAZ.
